# Type II Diabetes Mellitus in Arabic-Speaking Countries

**DOI:** 10.1155/2012/902873

**Published:** 2012-07-18

**Authors:** Mohammad Badran, Ismail Laher

**Affiliations:** Department of Pharmacology and Therapeutics, Faculty of Medicine, University of British Columbia, Vancouver, BC, Canada V6T 1Z3

## Abstract

The global epidemic of diabetes has not spared the Arabic-speaking countries, which have some of the highest prevalence of type II diabetes. This is particularly true of the Arab Gulf, a conglomerate of high income, oil-producing countries where prevalence rates are the highest. The prevalence rates among adults of the Arabic speaking countries as a whole range between 4%–21%, with the lowest being in Somalia and the highest in Kuwait. As economic growth has accelerated, so has the movement of the populations to urban centers where people are more likely to adopt lifestyles that embrace increased high-calorie food consumption and sedentary lifestyles. These factors likely contribute to the increased prevalence of obesity and diabetes in the Arabic speaking countries.

## 1. Introduction

Diabetes mellitus, long considered a disease of minor significance to world health, is now considered one of the main threats to human health in this century. The global epidemic of people with type II diabetes is largely due to population growth, aging, urbanization, and the scourge of obesity and physical inactivity. The total number of people worldwide with type II diabetes was expected to increase from 171 million in 2000 to 366 million in 2030 [1]. Unfortunately, the prevalence worldwide already reached 366 by 2011 according to the international Diabetes Federation (IDF), and the projections are that prevalence of diabetes on a global scale could well reach 530 million people in 2030 [1]. The World Health Organization (WHO) defines diabetes as a metabolic disorder of multiple etiologies characterized by chronic hyperglycemia with disturbances of carbohydrate, fat, and protein metabolism that results from defects in insulin secretion, insulin action, or both. Diabetes is associated with reduced life expectancy; the significant morbidity associated with diabetes arises from microvascular complications, increased risk of macrovascular complications (ischemic heart disease, stroke, and peripheral vascular disease), and diminished quality of life [2].

Type II diabetes-related mortalities account for 4.6 million deaths in 2011 for people aged 20–79 years, accounting for 8.2% of global all-cause mortality for people in this age group with an estimated rate of one death every seven seconds [3]. The number of deaths has increased by 13.3% from estimates for the year 2010 [4]. The magnitude of the estimated number of deaths due to diabetes is similar to the combined deaths from several infectious diseases like HIV/AIDS, malaria, and tuberculosis that are ranked as top public health priorities [3].

There is increased concern about the rising tide of type II diabetes and its associated complications in the Arabic-speaking countries (East Mediterranean, Arabic peninsula, and Northern Africa) as these regions have some of the highest rates of diabetes in the world [5]. This review considers the prevalence of diabetes in the Arab world and explores the contributing factors.

## 2. Diabetes Mellitus

Humans digest carbohydrates to form simpler sugars such as glucose, a monosaccharide that forms the primary carbohydrate energy source used by the body. In response to rising levels of blood glucose, pancreatic beta cells release insulin, the principal hormone that regulates uptake of glucose from the blood into most cells, where glucose is used either for generation of ATP, conversion to other molecules, or storage as glycogen and fat. Deficiency of insulin or insensitivity of its receptors can result in persistent or recurrent hyperglycemia, leading to a chronic noncommunicable disease called diabetes mellitus. The diagnostic criteria for diabetes as established by the WHO [6] are illustrated in Table 1. The Revisions for the 2010 Clinical Practice Recommendations now include the use of glycated hemoglobin (HbA1c) as a diagnostic criterion for diabetes, with HbA1c values ≥6.5% being diagnostic [7].

People with impaired fasting glucose (100–125 mg/dl (5.6–6.9 mmol/L)), and those with impaired glucose tolerance (at or above 140 mg/dl (7.8 mmol/L) but not over 200 mg/dl (11.1 mmol/L)), and with an HbA1c levels ranging between 5.7–6.4% are considered prediabetic and are at considerable risk for developing diabetes mellitus as well as cardiovascular diseases [8].

## 3. Risk Factors and Complications

The risk factors associated with type II diabetes can be grouped into two categories: modifiable and nonmodifiable risk factors. Modifiable risk factors include diets rich in saturated fats and simple carbohydrates, impaired glucose tolerance, metabolic syndrome, high blood pressure (≥140/90 mm Hg), elevated plasma triglycerides (≥250 mg/dl), and low levels of physical activity (<3 times a week). The nonmodifiable risk factors are age (older than 45 years), family history of diabetes, ethnicity, and diabetes during a previous pregnancy [9]. Diabetes can lead to serious complications if it is not properly managed: most of these complications are related to complications arising from microvascular (e.g., nephropathy, neuropathy, and retinopathy [10–12]) and macrovascular (e.g., coronary artery disease (CAD), peripheral artery disease (PAD), and cerebrovascular disease). Even though controlling blood sugar in diabetic patients is needed, treating high blood pressure and dyslipidemia is as important to prevent cerebrocardiovascular complications.

Diabetic patients have a 2–4 fold greater risk of developing CAD and PAD than nondiabetic individuals [13, 14], and the frequency of type II diabetes among patients presenting with stroke is 3 times more than that of matched controls [15]. The risk of stroke is increased by 150% to 400% for patients with diabetes, while the risk of stroke-related dementia increases by more than 3 fold in these patients [16–18]. Metabolic derangements such as hyperglycemia, dyslipidemia, and insulin resistance eventually lead to atherosclerosis through endothelial cell and vascular smooth muscle dysfunction combined with impaired platelet function and abnormal coagulation [19–21].

## 4. Prevalence of Diabetes

The prevalence of type II diabetes has increased dramatically in the Arabic-speaking countries over the last three decades, a trend that parallels increased industrial development. The wealth generated by oil-rich resources in countries of the Arabian Gulf have led to improved living standards, while there have also been accelerated urbanization, drastic changes in nutrition, reduced physical activity, and a greater reliance on mechanization and migrant workers.

As many as six Arabic-speaking countries are among the world's leaders in terms of type II diabetes prevalence: these countries are Kuwait, Lebanon, Qatar, Saudi Arabia, Bahrain, and United Arab Emirates (UAE).  Table 2 provides the 2010 International Diabetes Federation (IDF) statistics for type II diabetes prevalence in developed and developing countries. An estimated 9.1% of the populations from the Middle Eastern/North African region have type II diabetes (32.8 million) in 2011, and this is projected to reach 60 million in 2030. The explosion of type II diabetes in this region, within the 20–79 age groups, accounts for about 280,000 yearly deaths in the Middle Eastern/North African region, with mortality attributable to diabetes being equal in males (141,000) and females (138,000). About half of all diabetes-related mortality in this region occurs in people under the age of 60 years [3]. As for type I diabetes in the Middle Eastern/North African region, Saudi Arabia has the largest *number* of cases (65,000) of T1DM in children aged 0–14 years, while Kuwait has the highest *incidence* rate (22 cases per 100,000 per year) [3].

## 5. Factors Associated with Diabetes in Arabic-Speaking Countries

The outburst of diabetes in the Arabic-speaking countries is mainly due to type II diabetes. Although genetic factors likely play an important role in this epidemic, one cannot overlook the contributions made by the rapid development that has occurred in the last 30 years. Although such rapid economic growth brings with it great opportunities for improvements in infrastructure (e.g., health care and education), it also carries with it the burden of greater reliance on mechanization, a proliferation of Western-style fast food, access to cheap migrant labor, and as elsewhere, greater opportunities for sedentary lifestyles, especially in the young. These environmental factors fuel the emerging epidemic of type II diabetes in the Arabic-speaking nations, with these same factors also driving the current explosive increase in obesity in the Arabic-speaking regions [22].

### 5.1. Obesity

Obesity is the major risk factor for developing type II diabetes, as shown by the relationship between increases in body mass index (BMI) and the risk of developing type II diabetes [23–25]. Obesity is a strong predictor of type II diabetes in both genders and extends to all ethnic groups [26, 27]. An estimate from the National Center for Health Statistics (NHANES III) reported that 78.5% of diabetics were overweight, and 45.7% were obese. A meta-analysis of ten publications reveals an odds ratio of 2.14 for obese subjects developing type II diabetes [28].

There are several suggestions for why obese people can develop type II diabetes. Plasma leptin, nonesterified fatty acid, and tumor necrosis factor-*α* levels are all elevated in obese subjects and are thought to play essential roles in causing insulin resistance [27]. Data from the WHO shows that overweight and obesity prevalence has increased dramatically throughout recent decades in the Middle Eastern/North African region; importantly, this is accompanied by increased rates of type II diabetes. The prevalence of obesity in adults, adolescents, and children in the Middle Eastern/North African region is amongst the highest worldwide ranging between 2%–55% in adult females and 1%–30% in adult males while the prevalence in adolescents and children range from 5%–14% [29–34]. This adds to the economic burden associated with this malady since childhood obesity generally persists into adulthood: approximately one-third (26% male and 41% female) of obese preschool children and half (42% male and 63% female) of obese school-age children become obese at adulthood according to a survey of data collected between 1970–1992 [35].

A study from the US evaluated the relation between excess BMI years (a measure of the degree to which a person's BMI exceeds the reference BMI and the duration for which that person carries excess BMI) and incident diabetes, where participants were adolescents and young adults aged 14–21 years at the beginning of the National Survey of Youth 1979 and relied on self-reported measures of weight, height, and diabetes status (unspecified type) from 1981 through 2006. After excluding presumed type I diabetes, logistic regression was conducted to predict presumed type II diabetes as a function of sex, age, race excess BMI years and their specific interactions. An important finding was that for a given level of excess BMI years, younger individuals compared to older ones had higher risk of developing type II diabetes, meaning that a given amount of excess BMI when carried earlier in life may be more diabetogenic than the same amount of weight carried later in life. This indicates the necessity of weight-control interventions in the younger population of the Arabic-speaking countries to prevent or delay the incidence if type II diabetes [36].

### 5.2. Socioeconomic and Demographic Factors

**Figure 1:**
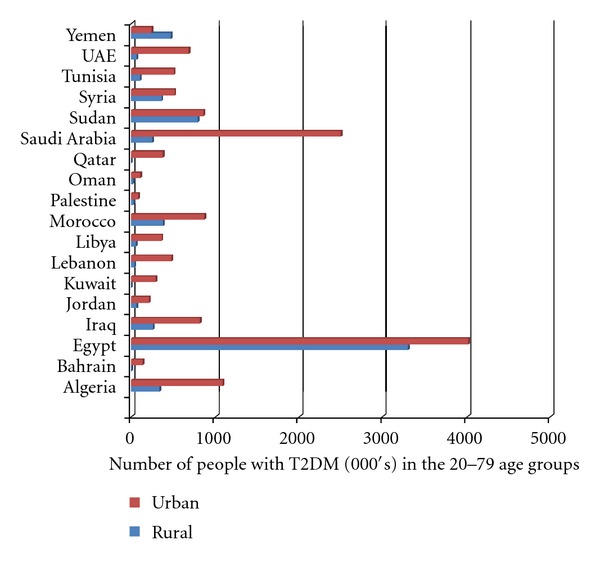
Prevalence of T2DM in urban and rural areas in the Arabic-speaking countries according to IDF estimates 2011 [3].

Modernization of Arab countries and the rapid development in large cities and towns increased urbanization of the population; an important difference between urban and rural sectors in the Arabic-speaking countries is the increased exposure to a more Western lifestyle [37]. In Saudi Arabia, 25.5% of the urban population is diabetic in comparison with 19.5% in rural areas. There are also regional differences in the prevalence of type II diabetes, with the Northern (27.9%) and Eastern (26.4%) provinces experiencing greater rates than the Southern region (18.2%), where a rural lifestyle is more common [38] and the population less prone to obesity than those on the Northern and Eastern provinces [39]. The ratio of people with type II diabetes in urban and rural areas is 235 to 100 in Oman [40] and 400 to 100 in Egypt [41].  Figure 1 shows the difference in type II diabetes prevalence between urban and rural areas in the Arabic-speaking countries.

The prevalence of type II diabetes in the Middle Eastern/North African region is greatest in the Arabian Gulf area, which are amongst the richest countries in the Arabic-speaking countries. The rate of type II diabetes in the low socioeconomic status (SES) population in urban areas in Egypt is 13.5%, which contrasts with a higher SES population in urban areas of Lebanon that has a prevalence of 20% [41]. Within Lebanon, the prevalence of type II diabetes is significantly lower in a high SES population (5%) compared to a low SES population (17%); this unexpected finding may be due to a lower mean age, shorter mean family history of type II diabetes, and a reduced mean body mass index (BMI) in the high SES Lebanese population [42]. Interestingly, those poor in rich society have higher type II diabetes than the rich in rich society or poor in poor society. Other studies show that low-income groups may be up to twice as prone to develop type II diabetes than high-income groups [43, 44], suggesting that income may be a weak determinant of type II diabetes prevalence in any given country.

The level of education allows increased awareness about type II diabetes risk factors, complications and management, and especially lifestyle choices [45]. A study of 3003 diabetic patients in Kuwait reported that 27.5% of diabetic patients were illiterate, while 15.5% were better educated [46]; similar findings were reported in Jordan and Qatar, where type II diabetes prevalence among the illiterate population was 34% and 23.5%, while among the university educated group was 7.7% and 11.3%, respectively [47, 48].

It is common for marriage to affect the lifestyle of couples from Arabic-speaking cultures as they become less active after marriage, tend to eat together, and likely reinforce increased food intake—so leading to the increased body weight observed in the Middle Eastern/North African region [49–52]. An important factor that can contribute to the increased prevalence of type II diabetes in the Arabic-speaking countries is the consanguinity in marriage [53]. In a study of married couples from Saudi Arabia, there is a positive correlation between consanguine marriages and type II diabetes, where 80% of all related marriages had a positive family history of type II diabetes as compared to 20% in nonrelated marriages [54]. This finding does not extend to other countries in the same region such as Israel/Palestine and Bahrain, where there is no significant increase in the prevalence of type II diabetes in the offspring of consanguineous mating [55, 56]. Further investigation is needed on the reasons for the variation in consanguinity and type II diabetes prevalence in Arabic-speaking countries.

### 5.3. Food Consumption

**Figure 2:**
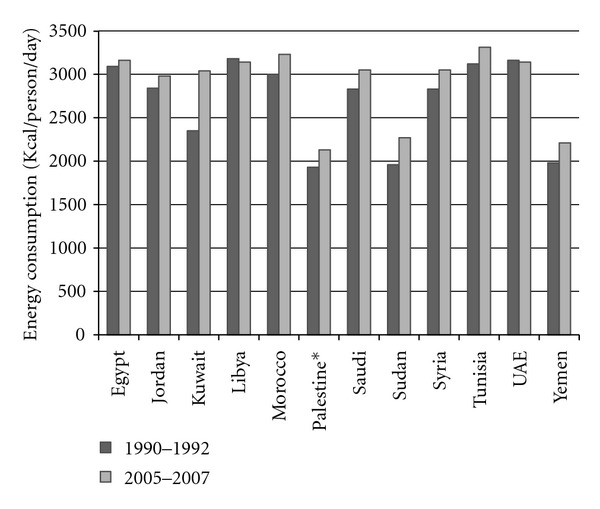
The dietary energy consumption per person, in kcal per day, for the period 1995–1997. Data are according to the Food and Agriculture Organization of the United Nations [66]. Energy consumption during 1995–1997. Kcal: A unit of measurement of dietary energy. One kcal equals 1,000 calories, and one kJ equals 1,000 joules.

The economic improvement and westernization in the Middle Eastern/North African region through the last four decades has resulted in drastic dietary changes, going from predominantly consuming dates, milk, fresh vegetables and fruit, whole wheat bread and fish to now mostly consuming foods rich in high saturated fats and refined carbohydrate diets coupled with a low dietary fiber intake [57]. These changes in dietary habits are associated with a steep rise in the prevalence of chronic diseases and obesity in the region [37, 58]. There are insufficient data on the relationship between lifestyle choices and diabetes prevalence in the Middle Eastern/North African region. Shown in Figure 2 is the dietary energy consumption per person in the Arab countries according to the Food and Agriculture Organization of the United Nations (Statistics Division) from 1990 to 2007, where the average energy consumption per person is 2780 kcal/day.

Some studies suggest that consumption of diets rich in saturated fats and poor in fiber are independent risk factors for T2DM [59–65]. Data from food balance sheets (1971–2005) confirm that energy intake per capita has increased in most of the Arabic-speaking countries, with increased contribution of fat to the daily energy supply (DES) being offset by lower consumption of carbohydrates as per the capita income increased [66]. In the Arabian Gulf, increased food intake is part of the socialization process and is a ritual based on large gatherings, where meals consisting of rice (high carbohydrates) and meat (high fat) are shared [39, 67]. In a recent study from Saudi Arabia, the adjusted odds ratio for eating Kabsa (a meal containing rice and meat) was 5.5, while that for vegetables was only 0.4 [68]. Even though people in Bahrain consume fresh fruit three times a week, they nonetheless eat fast food while watching TV [67]. Importantly, other high-income countries in Europe and North America actually consume more fat than Arabic-speaking countries, but nonetheless have lower rates of diabetes, [66], suggesting that other factors need to be considered when examining the role of diet in the prevalence of type II diabetes in the Middle Eastern/North African region.

### 5.4. Physical Inactivity

Physical activity is defined as any bodily movement produced by skeletal muscle that results in energy expenditure above basal levels [69]. A sedentary lifestyle increases the risk of developing type II diabetes and obesity [70, 71]. A study from the United States documented the relationship between physical activity, TV watching and the incidence of type II diabetes; not surprisingly, the least active men who watched TV more for than 15 hours per week had a significantly increased risk of type II diabetes (RR = 2.92) compared to men with high activity and less TV watching. An increment of 2 hours per day spent in watching TV was associated with 20% increase in risk of type II diabetes, while walking 40 minutes per day was associated with 19% reduction in risk [72]. The relative risk of type II diabetes was 16.75% for women who were obese and inactive compared to normal weight and active women [73]. As discussed earlier, the rapid economic development in the Arabic-speaking countries has resulted in significant changes in socioeconomic status and lifestyle; the extensive road networks, increased availability of cars, greater use of mechanized home and farm appliances, access to cheap migrant labor, widespread use of computers, televisions and electronic gaming devices has created an environment that fosters and encourages sedentary lifestyles. A Kuwaiti study reports that 58% of subjects with IGT were physically inactive compared to 4% who were vigorously active [46], while a Saudi study suggests that 81% of adult males in the city of Riyadh are inactive, with an astounding 99.5% of adult females of the Asir province also reporting no exercise of any intensity [74]. Likewise, only 2% of Egyptian adults exercise daily [75].

**Figure 3:**
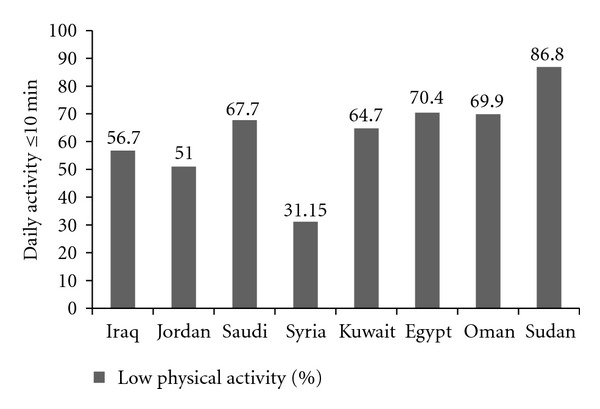
Percent of the population of selected countries of the Eastern Mediterranean Region reporting low levels of daily exercise. Data are according to the STEPwise survey [78].

Cultural barriers and limited access to sporting/exercise facilities are significant deterrents to engaging in physical activity in women of the Middle Eastern/North African region. These limitations are aggravated by the easy access to cheap migrant labor for domestic chores. For example, nearly all families in Kuwait and Saudi Arabia commonly employ cooks and maids—which encourages a sedentary lifestyle in indigenous women [67, 76]. Nearly half of the women in Palestine and Syria have sedentary lifestyles [49, 77], while in Bahrain, watching TV is the main leisure activity reported by women [52].  Figure 3 summarizes the percent of the population from various Middle Eastern/North African region engaging in daily physical activity lasting 10 minutes or less [78].

## 6. Diabetic Complications in Arabic-Speaking Countries

Among Saudi patients, there is a 31% prevalence of retinopathy in patients who had type II diabetes for at least 10 years [79], while data from the Western part of Saudi Arabia indicates that the prevalence of neuropathy in diabetic patients is about 82% (which is considered one of the highest in the world) with another 57% being asymptomatic [80]. Diabetic nephropathy is the major contributor to the need for dialysis in Saudi Arabia, where the number of diabetic patients entering renal replacement therapy increased dramatically from 4% in the early 1980s to 14.8% in the mid 1990s and shockingly to 40% in the late 1990s. The majority of deaths (60%) in patients entering dialysis are diabetic patients [81, 82]. About 37–41% of diabetic patients in Saudi Arabia develop a stroke [83, 84], while 61% of diabetic patients have peripheral artery disease [85].

In a cross-sectional study from Egypt, 42% of diabetic patients had nephropathy, 22% had peripheral neuropathy, 0.8% had foot ulcers, and 5% were blind [86]. In Oman, prevalence of diabetic retinopathy was 14.4% [87], while in Yemen it reached 55% [86]. In Jordan, 45% of diabetic patients at a national diabetes center had retinopathy, 33% had nephropathy, and 5% had amputations [88]. At a diabetic clinic in Libya 30% of patients had retinopathy, 25% had nephropathy, and 45% had neuropathy [89].

In addition to micro- and macro-vascular complications, type II diabetes also affects the mental health of patients. In Sharjah, UAE, a cross-sectional study was performed to estimate the prevalence of psychological distress and its correlates in diabetic patients. Patients were interviewed using a structured questionnaire to assemble data on lifestyle factors, sociodemographics, medication usage, and diabetic complications. The K6 was used as a screening tool for mental health concerns (K6 is a six-item self-reporting scale that assesses the frequency with which an individual experiences symptoms of general psychological distress, e.g., nervousness, tiredness, hopelessness, and restlessness; these are frequently associated with mental illness). The score for each item ranges from 1 (“none of the time”) to 5 (“all of the time”). Summing the unweighted items yields a total score that ranges from 6 to 30, with scores of 19 and over indicating possible mental illness. Approximately 12.5% of diabetic patients scored 19 and higher, indicating possible mental health concerns. The K6 scores were significantly higher in patients with diabetic complications such as retinopathy and lower limb neuropathy. Thus diabetic complications can affect mental health status, where patients with psychological distress have a greater severity of physical symptoms, poorer self-care and decreased compliance to medications, suggesting that improving the mental health status of diabetic patients could provide significant improvements to the long term outcomes of these patients [90].

## 7. Cost of Diabetes

The methodological approach used to estimate the costs of diabetes have varied. Early studies used the designs of Rice [91, 92] and examined data by the International Classification of Diseases (ICD) method [93]. More recent studies rely on complex designs that merge the concepts of the top-down and bottom-up approaches and examining costs due to comorbidity. Studies that estimate the cost of diabetes can be categorized into three study designs: (i) based on category data (ICD codes) from general population surveys [94], (ii) cost projections from previous studies [95], and (iii) responses from persons with diabetes [96].

**Figure 4:**
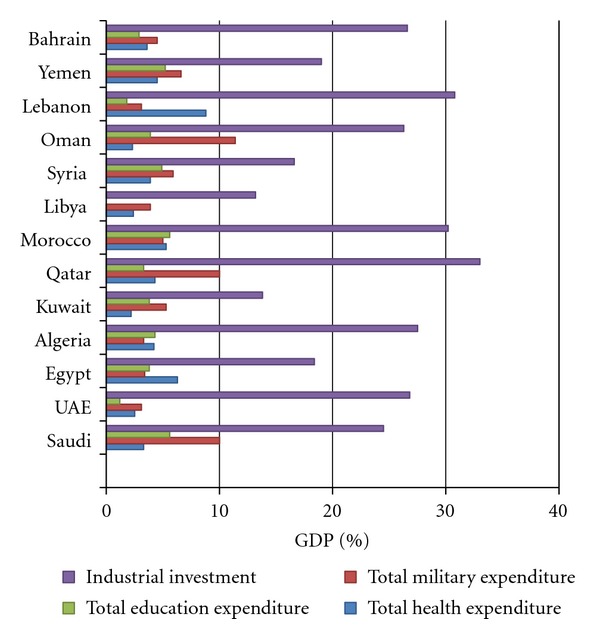
Industrial investment, military, education, and health expenses as a percentage of GDP in selected Arabic-speaking countries. GDP: gross domestic product. Sources: health expenditure [97], industrial investment, education, and military expenditure [98].

Although the Middle Eastern/North African region reports extremely high numbers of type II diabetes patients, it is estimated that only USD 10.9 billion is spent on diabetes-related costs as to the USA (USD 223 billion) and Europe (131 billion USD), who spend proportionately more on diabetes related expenses [3]. As shown in Figure 4, most of the Arabic-speaking countries spend less than 7% of their GDP on health care systems and only 6% on education, while spending 19–33% on industrial investment, and outspend all other nations by allocating 11% of the GDP to military related expenses, [97, 98]. One can only speculate why health care systems in the Arabic-speaking countries are underresourced and perform below expectations: the chain of command consists of inefficient bureaucrats with political objective that are often at odds with public health and wellbeing, and where health professionals and support systems are largely concentrated in urban areas [99].

## 8. Prevention of Diabetes

The adage that prevention is better than cure holds true for every society and circumstance. Using a simple questionnaire that assesses easily available information such as family history, cardiovascular risk factors, age, and waist-to-hip circumference ratios is an easily implementable first step for identifying people at risk for developing type II diabetes. Once identified, such individuals should undergo routine and inexpensive careening tests for diabetes such as measuring levels of plasma and urine glucose, fasting glucose levels and possibly undergo a glucose tolerance test, especially when type II diabetes is strongly indicated [3]. 

type II diabetes can be prevented or its progression impeded by implementing lifestyle changes. Modifications of lifestyle have no costs associated with it, are usually free of side effects, and can be as (or even more) beneficial than some pharmacological approaches [100]. These lifestyle changes are often designed to reduce obesity, a state promoted by increased energy intake and low levels of physical activity [101, 102]. Importantly, current guidelines suggest only modest but regular exercise as being a beneficial strategy in the management of type II diabetes [103].

Several randomized clinical trials confirm that diet and exercise can decrease the incidence of type II diabetes. The Da Qing Diabetes Prevention Study in China reports, that had a 6-years follow-up period reported, that only 47% of the patients in the diet plus exercise group had type II diabetes, while 68% of patients in the control group were diabetic; diet and exercise alone produced a 42% risk reduction for developing type II diabetes [104]. A follow-up study at the 20-years follow-up stage indicated that there remained a 43% lower incidence of type II diabetes in those patients who were part of the diet plus exercise group [105]. Similar findings were also reported in the Finnish Diabetes Prevention Study that randomly assigned 522 middle-aged, overweight men and women with IGT into either an intensive lifestyle intervention group or a control group. After an average of 3.2 years, the incidence of type II diabetes in the control group was twice as great as that in the intervention group (23% versus 11%), while the risk of type II diabetes was reduced by 58% through lifestyle modification [106]. These data are supported by similar findings from the United States and India [107–109].

## 9. Future Directions

Appropriate use of screening tools is the most important factor in preventing and controlling type II diabetes. For example, a study of Arab-Americans found that using A1C alone as a screening tool resulted in high proportion of false-negative test results for both prediabetics and diabetics, which could well result in delayed diagnosis and progression of diabetes-related complications [110]. Thus, using FPG and/or OGTT seems to be more effective in assessing glucose tolerance in Arabs.

In Oman, a simple diabetes risk score was developed to identify individuals with high risk of developing type II diabetes; the score had high sensitivity and specificity in two cohort studies and was able to identify most individuals at high risk of developing type II diabetes in community-based settings. Of interest is that the Thai, Dutch, Finnish, and Danish diabetes risk scores performed poorly in Omani Arabs [111], indicating that a customized diabetes risk scoring system may be better suited for patients of Arabic-speaking descent. The diabetes risk score developed in Oman is inexpensive and easy to self-administer and may be a first step in for screening in Arabs at high risk of developing type II diabetes.

Insulin secretion and sensitivity after oral glucose tolerance test (OGTT) differs in different ethnic groups. A study comparing normal glucose tolerance (NGT) and impaired glucose tolerance (IGT) where subjects from different ethnic backgrounds (seven hundred and eighteen subjects of Arabs, Japanese, and Mexican-American decent) received an OGTT and had plasma glucose and insulin levels recorded every 30 min and used the Matsuda index of insulin sensitivity. Regardless of their ethnic backgrounds, patients with IGT had both impaired insulin secretion and sensitivity compared to NGT subjects. However, reduced insulin secretion was the highest in Arabs (80%), while lower in Japanese (55%) and Mexican-Americans (41%). Conversely, decreased insulin sensitivity was greatest in Mexican-American (30%), lowest in Arabs (11.5%), and intermediate in Japanese (23%) [112]. These data suggest that diabetic Arab patients suffer from impaired insulin secretion to a greater extent than from decreased insulin sensitivity and imply that therapeutic choices and management for diabetic patients may require revised criteria in patients from the Arabic-speaking countries. It would be useful if such studies considered different ethnic Arabic groups (Arabs from African descendant versus Arabs from Caucasian descendant).

Other examples of ethnic based-disease dynamics come from a study indicating that that the majority of Asian-Indians patients with acute coronary syndrome without a history of type II diabetes nonetheless have shown hyperglycemia. Short-term follow-up studies indicated that almost two-thirds of these subjects continued to have abnormal glucose metabolism and increased insulin resistance [113]. Similar studies of CAD are lacking in Arabic-speaking countries. It is known that healthy South Asians can develop type II diabetes and myocardial infarction at younger ages and lower BMI values compared to Europeans [114–116], likely due to a higher total body fat and greater ectopic fat deposition in the liver, abdomen, and elsewhere and lower storage capacity of subcutaneous adipose tissue. As a result, South Asians have higher adipocyte surface areas and increased secretions of tumor necrosis factor *α* and free fatty acids, both of which can lead to insulin resistance [117]. Similar studies are lacking in Arabic-speaking diabetics to clarify whether such patients are increased risk of insulin resistance and cardiovascular disease relative to the levels of non-Arabic patients who have similar BMI's, HbA1c, and so forth.

## 10. Conclusion

Type II diabetes is an emerging epidemic of titanic proportions in the Arabic-speaking countries, threatening to undermine the benefits of modernization and economic resurgence. Several socioeconomic, dietary, and lifestyle factors are associated with type II diabetes in these countries. However, there is an urgent need for comprehensive studies on the role of these factors and their contribution in the occurrence of type II diabetes. Most studies are cross-sectional with limited sample sizes that in most cases cover only some parts of the country. Further studies on the roles of diet and physical exercise and their relationships with increased type II diabetes prevalence are needed in the Arabic-speaking countries.

Limited spending on health care and lack of education (formal and informal) continue to be pervasive even though there are great strides being made in industrial and military empowerment. There continues to be inadequate public awareness of healthy eating habits and of the interactions of diet, exercise, and chronic diseases. It would be particularly important to develop programs for early-age exercise programs and nutrition education, regardless of regional or gender issues since Arabic-speaking countries suffer from poor exercise, which is probably the main reason for such high diabetes and obesity prevalence. It is very likely that healthy practices related to the prevention and management of type II diabetes can easily be implemented in ways that do not conflict with cultural norms of the Arabic-speaking countries.

## Figures and Tables

**Table 1:** Diagnostic criteria for diabetes according to the WHO [6].

Condition	2-hour glucose mg/dL (mmol/L)	Fasting glucose mg/dL (mmol/L)
Normal	<140 (<7.8)	<110 (<6.1)
Impaired fasting glucose	<140 (<7.8)	≥110 (≥6.1) and <126 (<7)
Impaired glucose tolerance	≥140 (≥7.8)	<126 (<7)
Diabetes mellitus	≥200 (≥11.1)	≥126 (≥7)

**Table 2:** Ranking of the prevalence of type II diabetes in Arabic- and non-Arabic-speaking countries. The data are separated for males and females aged between 20–79 years, using IDF estimates for 2011. Population numbers are taken from the CIA World Factbook 2008. Arabic speaking countries are shown in bold.

Country	Population (×1,000)	Comparative^∗^ diabetes prevalence (%)	Male (×1000)	Female (×1000)
**K** **uwait**	**1,868**	**21.2**	**175.3**	**122.6**
**Lebanon**	**2,788**	**20.1**	**230.9**	**296.9**
**Qatar**	**1,541**	**20.1**	**166.2**	**50.6**
**Saudi Arabia**	**17,023**	**20.0**	**1,450.7**	**1,308.8**
**Bahrain**	**986**	**19.8**	**91.4**	**59.3**
**UAE**	**6,107**	**19.2**	**296.2**	**128.9**
**Egypt**	**48,305**	**16.9**	**3,123.7**	**4,199.5**
Mexico	69,323	15.8	5,457.1	4,836.6
**Libya**	**3,875**	**14.1**	**211.2**	**225.1**
**Jordan**	**3,268**	**12.3**	**148.8**	**142.7**
**Oman**	**1,810**	**10.7**	**88.1**	**50.3**
**Syria**	**10,824**	**10.1**	**437.1**	**452.3**
Russia	109,166	10.0	5,227.4	7,365.6
Cypress	809	9.1	56.5	25.3
**Yemen**	**10,902**	**9.8**	**366.1**	**361.1**
**Tunisia**	**7,084**	**9.6**	**278.3**	**351.2**
USA	216,804	9.5	11,986.2	11,735.5
**Iraq**	**15,068**	**9.3**	**459.1**	**629.9**
**O** **P** T*	**1,896**	**9.3**	**51.8**	**72.6**
China	968,974	9.0	50,293.2	39,751.8
**Sudan**	**22,000**	**8.7**	**947.9**	**718.7**
Canada	25,140	8.6	1,479.7	1,236.4
India	737,003	8.3	32,498.1	28,760.3
Turkey	47,322	8.1	1,469.7	2,033.1
Italy	45,637	7.8	1,734.8	1,825.5
Israel	4,707	7.6	206.2	194.1
**Algeria**	**22,619**	**7.0**	**704.4**	**730.7**
**Morocco**	**19,964**	**6.9**	**608.7**	**659.1**
**Djibouti**	**480.9**	**6.4**	**13.1**	**13.1**
Germany	62,810	5.5	2,674.2	2,347.9
France	44,328	5.5	1,733.8	1,503.7
UK	44,813	5.3	1,790.1	1,273.8
**Mauritania**	**1,756**	**4.3**	**29.1**	**31.4**
**Somalia**	**4,275**	**4.2**	**87.6**	**97.4**

*All comparisons should be done using the comparative prevalence, which is adjusted to the world population.
